# Design and rationale of the HCC BRIDGE study in China: a longitudinal, multicenter cohort trial in hepatocellular carcinoma

**DOI:** 10.1186/1471-230X-11-53

**Published:** 2011-05-12

**Authors:** Minshan Chen, Terry Therneau, Lucinda S Orsini, You-Lin Qiao

**Affiliations:** 1 Department of Hepatobiliary Surgery, Sun Yat-Sen University Cancer Center, Guangdong Province, China; 2 Division of Biomedical Statistics and Informatics, Mayo Clinic, Rochester, Minnesota, USA; 3 Bristol-Myers Squibb, Wallingford, Connecticut, USA; 4 Department of Cancer Epidemiology, Cancer Institute & Hospital, Chinese Academy of Medical Sciences & Peking Union Medical College, Beijing, China

## Abstract

**Background:**

More than 50% of the worldwide cases of hepatocellular carcinoma occur in China, and this malignancy currently represents the country's second leading cause of cancer death in cities and the leading cause in rural areas. Despite recent advances in the control and management of hepatocellular carcinoma within China, this disease remains a major health care issue. The global HCC BRIDGE study, designed to assess patterns of hepatocellular carcinoma therapy use and associated outcomes across real-world clinical practice, has recently been expanded as a national study in China, allowing a detailed analysis of hepatocellular carcinoma in this important country.

**Methods/Design:**

The global HCC BRIDGE study is a multiregional longitudinal cohort trial including patients newly diagnosed with hepatocellular carcinoma between January 1, 2005, and June 30, 2011, who are receiving treatment for hepatocellular carcinoma via sites in the Asia-Pacific, European, and North American regions. The HCC BRIDGE China national study comprises the portion of the global HCC BRIDGE study conducted within mainland China. Patients will be followed from time of diagnosis of hepatocellular carcinoma (post-January 1, 2005) to time of death or December 31, 2011, whichever comes first. Data will be collected on demographic/clinical characteristics, relevant laboratory values, hepatocellular carcinoma/underlying liver disease treatment, tumor response, adverse events, hospitalizations, and overall survival. The primary study end point is overall survival; secondary end points are disease progression, treatment-limiting adverse events, and treatment failure.

**Results:**

At the time of writing, 15 sites have selected for participation across all 7 traditional regions of China (North, North-East, East, South, South-West, North-West, and Central). The anticipated study population from the China national study is approximately 9000 patients.

**Discussion:**

Findings from the HCC BRIDGE China national study, the first geographically representative study of hepatocellular carcinoma in China, will contribute to the understanding of patterns of therapy use and related clinical outcomes and will provide further information on continuing unmet needs for hepatocellular carcinoma throughout this important country.

## Background

Hepatocellular carcinoma (HCC), one of the world's most prevalent and deadly malignancies [1,2], is particularly common in Asia-Pacific countries, with more than half of all cases occurring in China alone [2,3]. In most patients, HCC develops in association with liver cirrhosis and/or viral hepatitis infection. Indeed, the main risk factors for the development of HCC among the Chinese population are the presence of liver cirrhosis and chronic infection with hepatitis B virus [4]. Other risk factors include chronic hepatitis C virus infection, a family history of liver cancer, depression, ingestion of aflatoxin B1 via contaminated foodstuffs, and excessive alcohol consumption [4,5].

Current therapeutic options for HCC in China are varied, with treatment allocation generally dependent on the patient's liver function and tumor burden. Commonly used therapies include surgical techniques, such as liver resection and liver transplantation; interventional therapies, such as hepatic artery infusion and transcatheter arterial chemoembolization; and ablation techniques, such as anhydrous alcohol injection and radiofrequency or microwave ablation [6]. Systemic therapy options, including radiotherapy, immunotherapy, standard chemotherapy, and traditional Chinese herbal medicines, are also recommended [6]. In addition, the multikinase inhibitor sorafenib, which has demonstrated a reduction in death risk among advanced, inoperable HCC patients with mostly Child-Pugh A cirrhosis [7,8], is approved in China for treatment of advanced unresectable HCC.

In recent years, the China Ministry of Health has made great strides in the management of HCC by establishing it as one of 5 tumors of high national importance and by initiating a number of primary HCC prevention activities. These include providing government-funded HCC screening for persons in high-risk areas (within the Jiangsu and Guangxi provinces) who are positive for hepatitis B surface antigen and who have cirrhosis and, more recently, enacting mandatory hepatitis B virus vaccination programs. Despite these advances, HCC remains a cancer with a high mortality rate and a clear unmet medical need in China. Based on data from the Third China National Mortality Survey conducted in 2004/2005, HCC is the second leading cause of cancer mortality in cities and the leading cause in rural areas [9]. Moreover, this survey reported an HCC mortality rate in China of 26 deaths per 100,000 persons. When considering these mortality data, it is important to acknowledge that they are pooled from individual studies supported by the China Ministry of Health, and only represent certain regions of the country. As such, the reported national mortality rate may not accurately reflect the true burden of HCC across the country. Furthermore, given the lack of active surveillance, there remain many unanswered questions surrounding the epidemiology and management of HCC in China.

The global HCC BRIDGE study is an ongoing longitudinal cohort trial of patients with HCC in 3 core geographic regions (Asia-Pacific, European, and North American) that is designed to (1) evaluate current treatment approaches to HCC and associated clinical outcomes, and (2) determine the real-world impact on clinical outcomes of new HCC treatments, such as sorafenib, versus other therapies [10]. Recently, the HCC BRIDGE study was expanded as a national study in China allowing a specific and more detailed evaluation of HCC across this important country.

## Methods

### Study design

The global HCC BRIDGE study is a multiregional longitudinal cohort trial including patients diagnosed with HCC from January 1, 2005, to June 30, 2011 [10]. Patients enrolled in the study will be followed from the date of HCC diagnosis to December 31, 2011, or date of death, whichever comes first. The HCC BRIDGE China national study comprises the portion of the global HCC BRIDGE study conducted within mainland China. To ensure that the national study is geographically representative, it was designed to include patients enrolled at sites from all 7 traditional regions across China (North, North-East, East, South, South-West, North-West, and Central).

### Study sites

Sites have been selected for inclusion in the China national study on the basis that (1) they are tertiary care centers providing surgical and routine follow-up care for patients with HCC or are oncology centers that care for these patients; (2) the type and proportion of etiologies for HCC are consistent with the Chinese national average; and (3) the HCC screening practices, when used, are in accordance with Chinese national standards. In addition, sites may be selected if the patient population was, on a prior occasion, used to characterize the Chinese national HCC population for other research purposes, for example during evaluation of national HCC incidence/prevalence rates or the establishment of national HCC staging systems.

### Patients

All patients enrolled in the HCC BRIDGE study must meet 3 key inclusion criteria: (1) diagnosed with HCC between January 1, 2005, and June 30, 2011, in accordance with American Association for the Study of Liver Diseases/European Association for the Study of the Liver guidelines [11]; (2) aged at least 18 years at the time of HCC diagnosis; and (3) received or receiving treatment for HCC through a site enrolled in the HCC BRIDGE trial. Patients participating in other clinical trials will be included in the study if the patient is enrolled in a single-arm clinical trial, enrolled in a randomized clinical trial of HCC adjuvant therapy, or was not selected to receive active treatment in a randomized clinical trial that was conducted between 2005 and 2007. Any patient whose first active HCC-directed treatment is received via participation in a randomized clinical trial of primary HCC therapy will be excluded from the current study, since this treatment regimen would have been determined by a study protocol rather than by the treating physician. Similarly, patients included in the China national study who subsequently enroll in such a trial will be censored from the study at that time point. Patients with unknown month and year of HCC diagnosis or of the first visit at the site of inclusion will be excluded. Furthermore, to preserve regional representation within China, patients who reside outside the province of interest for a particular study site will also be excluded. Finally, in order to avoid selection bias when the number of eligible patients at a site exceeds the number allowed according to study sample size calculations, an enrollment scheme will be adopted such that the same number of consecutive patients are enrolled each month. For example, in the first year of data entry, if a site is to enroll 200 patients, approximately 17 consecutive patients diagnosed with HCC will be enrolled in each month. If there are less than the specified numbers of patients in any given month, we ask that the corresponding number of extra patients be enrolled in the following month.

### Comparator groups

In order to evaluate the influence of sorafenib on HCC treatment patterns in China, clinical outcomes will be compared across 2 periods within the overall study duration: (1) the period when sorafenib was unavailable (2005 to 2007) and (2) the period when sorafenib was available for use (2008 to 2011). These 2 periods will be differentiated by the first record of sorafenib use at each site. Patients treated during the period when sorafenib was not available will act as historic controls and will be statistically matched within the pool of patient data by age, sex, Child-Pugh status, and HCC stage to patients treated with sorafenib between 2008 and 2011. Patients treated with non-sorafenib therapies during the period when sorafenib was available will act as concurrent controls and will be used to evaluate characteristics of HCC patients treated with sorafenib versus other therapies. In this study, "sorafenib treatment" is defined as the use of either sorafenib alone or concomitant use of sorafenib and other pharmacologic agents or interventions within 21 days of each other.

### End points

The primary end point will be overall survival (all-cause mortality); secondary end points will include disease progression, treatment-limiting adverse events, and treatment failure (Table 1). Data from these protocol-defined end points will be used to analyze treatment patterns and therapeutic outcomes as described in the following sections.

**Table 1:** Definitions of study end points

**Primary end point**
Overall survival
Number of days from date of treatment initiation to date of death. Death will be verified by sites based on death certificates or via caregiver, relative, or health care provider, and death information source will be captured in data collection. Surviving patients will be censored from last date of treatment assessment.
**Secondary end points**
Disease progression
Number of patients by treatment intervention with MRI/CT evidence of visible arterial enhancement, radiologic report of progression, or radiologic report of new tumor growth or symptomatic/clinical progression.
Time to disease progression
Number of days from date of treatment initiation to date of documented disease recurrence based on MRI or CT by treatment intervention, or symptomatic/clinical progression. Patients without recurrence will be censored from last date of treatment assessment.
Complication rate
Number of patients by treatment intervention who were withdrawn due to adverse events prior to introduction of other therapy.
Time to treatment-limiting adverse events
Number of days from first date of treatment to date of withdrawal due to adverse event(s) prior to introduction of other therapy. Patients without treatment-limiting adverse events will be censored at last date of treatment assessment.
Treatment failure rate
Number of patients by treatment intervention with a finding of treatment switch, HCC-related hospitalization and emergency room visit, disease recurrence, or death prior to introduction of other therapy.
Time to treatment failure
Number of days to first treatment failure overall and by individual item. Patients without treatment failure will be censored from last date of treatment assessment.

CT, computed tomography; MRI, magnetic resonance imaging.

### Data collection

The following data will be systematically collected for all enrolled patients: (1) date of diagnosis and/or date of first HCC visit; (2) demographic and clinical characteristics at the time of diagnosis/first HCC visit and at all occurrences of new treatment intervention during the study period via medical chart review; (3) data on variants of HCC diagnosis, for example, mixed HCC, fibrolamellar HCC, and on the additional diagnosis of cholangiocarcinoma; (4) data on the use of currently available HCC treatment approaches during the study period from medical chart review; (5) data on the use of new treatments for HCC if they become available and are used prior to study end; (6) data on hospitalizations, clinical visits, and visits to other health care professionals, with collection of hepatic function, tumor assessments, and laboratory values according to clinical judgment; and (7) data on method of payment for health care utilization. All data will be collected retrospectively and prospectively at study sites in order to account for patient records over the full duration of the study; data collection is expected to be complete by June 2012. All sites will enter data into an electronic data capture system developed by Outcome Sciences, Inc. (Cambridge, MA, USA). Outcome Sciences will manage the data system, including monthly data monitoring and cleaning, site queries, and check of invalid clinical values determined a priori by study investigators. All data collection procedures will comply with Chinese privacy and confidentiality requirements.

### Sample size

Sample size requirements for the Chinese regional sites are approximately 200 patients per site per year between 2008 and 2010 (plus approximately 192 per site for the first 6 months of 2011) to capture current treatment and clinical outcomes in HCC, and 100 patients per site between 2005 and 2007 for historic controls. Pooling of data across all sites in China will be employed to describe disease and treatment characteristics across the country.

### Data analysis

To evaluate therapeutic approaches to HCC in China, the number and percentage of patients using each treatment (plus any new treatments that are approved and used within the study period) will be determined for each study year, in total and stratified by disease stage and site. Treatment patterns will also be assessed by disease stage for each study year. Patient characteristics will be stratified by disease stage and by site for each study year.

To assess therapeutic outcomes, age- and gender-adjusted rates will be calculated for each study end point. Cox proportional hazard models will be used for the time-to-event end points (overall survival and disease progression) to determine hazard ratios for each treatment approach, and Kaplan-Meier curves will be generated. Logistic regression models will be used for categorical end points (treatment-limiting adverse events and treatment failure). Covariates for the models will include disease stage, gender, age, duration of disease, etiology of disease, liver status, tumor size/spread, surgical intervention/number of interventions, and number of comorbidities; analyses will be conducted at the site and country level. The same analysis plan will be adopted for the evaluation of survival and other clinical end points with sorafenib versus other therapies, except that "treatment" will be included as a covariate. Further analyses will be conducted to evaluate clinical characteristics and demographics of patients treated with sorafenib versus other treatments for HCC. In addition, data collected will be used to analyze additional aspects related to HCC epidemiology and management in China (Table 2). All tests of statistical significance will be carried out at the 0.05 level.

**Table 2:** Additional aspects of HCC management for potential evaluation using data from the HCC BRIDGE China national study.

**Outcomes in patient subpopulations**
• Assessment of treatment use and clinical outcomes among patients with:
◦ Traditional HCC risk factors, such as hepatitis B or hepatitis C virus infection (or co-infection), alcoholic liver disease, or cirrhosis
◦ Diabetes or multiple metabolic disorder
◦ HIV co-infection
• Assessment of the impact of underlying disease management on clinical outcomes in these patients
• Evaluation of potential regional/provincial differences in outcomes among patient subpopulations
**Impact of HCC screening**
• Assessment of influence of screening on:
◦ Treatment allocation and clinical outcomes
◦ Overall survival
◦ Underlying disease
• Evaluation of the cost-effectiveness of mandatory HCC screening
**Health care access**
• Evaluation of the level of health care access in different regions/provinces and association with clinical outcomes
• Examination of any potential influence of immigrant/minority status, risk factor status, and alcohol and drug use on health care access

### Loss to follow-up

Throughout the HCC BRIDGE China national study, patients are expected to leave via loss to follow-up, by consenting to enter a randomized clinical trial of primary HCC treatment, or at death. Death certificate searches or interviews with the patient's caregiver, relative, or health care provider will be used to verify deaths. The inability to verify deaths is expected for 5% of patients in the study. Sample size calculations have been adjusted accordingly to account for this level of missing data for the primary end point of the study.

## Results

**Figure 1:**
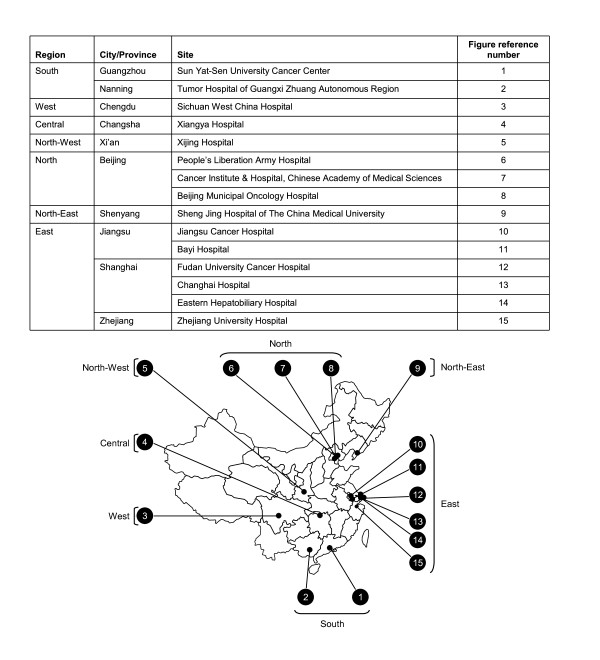
**Geographic distribution of sites currently included in the HCC BRIDGE China national study**.

The number of patients enrolled across the various geographic regions captured in the global HCC BRIDGE study is expected to exceed 20,000. For the HCC BRIDGE China national study, a total of 15 sites across China have been selected for inclusion at the time of writing. At present, the anticipated country-wide study population is approximately 9000 patients (Figure 1).

## Discussion

Over the past 3 to 4 decades, China has experienced a substantial increase in the incidence of HCC [5], and the country now accounts for more than half of the world's cases of this malignancy [2,3]. Nevertheless, in spite of the huge health care burden of HCC in China, there has never been a truly geographically representative study of HCC in this country and active surveillance is currently lacking. This suggests that the current estimates of the incidence and prevalence of HCC in China may not be accurate, and that regions with a current high unmet need and/or a high risk for new HCC cases may be unidentified.

The HCC BRIDGE China national study is the first geographically representative epidemiologic study of HCC in China and is expected to include more than 9000 patients over its course. This study, via its inclusion of a large number of sites across all 7 traditional regions of China, will facilitate a thorough assessment of HCC patient characteristics, treatment allocation, and clinical outcomes, across the entire country, as well as allowing a unique analysis of possible regional variations in these aspects of HCC epidemiology and management. Ultimately, the study will help determine levels of unmet medical need and identify regions of high risk for HCC within China.

As well as contributing to the general understanding of treatment patterns and associated outcomes among HCC patients in China, the data generated by the HCC BRIDGE China national study may provide insights into other aspects of HCC epidemiology and management, such as the potential role of HCC screening in treatment allocation and outcomes, and possible differences in clinical outcomes among different HCC patient subpopulations. Moreover, the study data may further help address other unanswered questions related to HCC in China, including questions about the appropriateness of current standards of care for HCC, the extent of usage and the impact of traditional Chinese herbal medicine, and best practices in clinical care for Chinese patients with HCC. Through these investigations and analyses, the study also has the potential to inform ongoing China public health reform, policy, and practice.

The eventual findings of the HCC BRIDGE China national study will need to be considered in light of the study's strengths and weaknesses. The primary strengths of this study are (1) the large number of patients included; (2) the geographic representativeness of the included sites; and (3) the use of real-life clinical practice data. Additional strengths include the use of centralized data monitoring, cleaning, site queries, and checking of invalid clinical values, which will enhance the quality of the collected data, as well as the use of identified prognostic factors for HCC survival to control for population differences that may influence treatment outcomes. The main potential study limitations are (1) treatment comparisons are performed in patients without prospective randomization methodology; (2) both academic and nonacademic centers/hospitals are included, which may result in differing quality of medical care for enrolled patients; (3) data quality is dependent on the thoroughness of the clinician's documentation of medical history, treatment, and outcomes; and (4) data collection bias is possible since clinical assessments are not conducted at predefined time points, as in a clinical trial, but at times determined by clinicians and their patients.

## Conclusion

In conclusion, the HCC BRIDGE China national study represents the first geographically representative study of HCC in China and will contribute significantly to the understanding of patterns of HCC therapy use and related clinical outcomes, as well as knowledge of continuing unmet needs for HCC.

## Competing interests

**MC: **Minshan Chen declares that he has no competing interests

**TT: **Terry Therneau declares that he has no competing interests

**LO: **Lucinda Orsini is currently an employee of Bristol-Myers Squibb

**YLQ: **You-Lin Qiao declares that he has no competing interests

## Authors' contributions

**MC **participated in the design of the study, contributed to the statistical analysis plan and was involved in the drafting of the manuscript, revising for important intellectual content, and fully approved the final version for submission. **TT **contributed to the statistical analysis plan and participated in the oversight of the study during data acquisition, and was also involved in the drafting of the manuscript, revising for important intellectual content, and fully approved the final version for submission. **LO **conceived of the study and participated in its design and coordination and was involved in the drafting of the manuscript, revising for important intellectual content, and fully approved the final version for submission. **YLQ **conceived of the study and participated in its design and coordination, and was also involved in the drafting of the manuscript, revising for important intellectual content, and fully approved the final version for submission.

## Acknowledgements and funding

The authors would like to acknowledge the essential contribution of the other clinical investigators participating in the HCC BRIDGE China national study, as follows: Lequn Li, JiJin Jiang, Guohong Han, Enshua Xiao, Lvnan Yan, Yuxian Bai, and Ping Zhao. The authors would also like to thank Li Ling (EXCEL); Lindsay Dudgeon (Outcome Sciences, Inc.); and Gabriella Cucinotta, Hannah Chen, Helena Zhu, and Wenjun Xiao (Bristol-Myers Squibb) for various study and data management activities. During the development of this manuscript, editorial support was provided by Richard Daniel, PhD, of PAREXEL, and was funded by Bristol-Myers Squibb. The HCC BRIDGE study is supported by research funding from Bristol-Myers Squibb (Study No. CA182023).

## Pre-publication history

The pre-publication history for this paper can be accessed here:

http://www.biomedcentral.com/1471-230X/11/53/prepub
